# Patients’ Shift from Public to Private Primary Health Care Providers in Lithuania: Analysis of the Main Reasons

**DOI:** 10.1177/00469580211060304

**Published:** 2021-12-20

**Authors:** Paulius Žvinakis, Roberta Kalibataitė, Vytenis Kalibatas

**Affiliations:** 1 Department of Health Management, Faculty of Public Health, Lithuanian University of Health Sciences, Kaunas, Lithuania; 2 Department of Dental and Oral Pathology, Faculty of Odontology, 475578 Lithuanian University of Health Sciences, Kaunas, Lithuania; 3 Department of Health Management, Faculty of Public Health, Lithuanian University of Health Sciences, Kaunas, Lithuania

**Keywords:** primary health care, family physicians, public sector, private sector, patient acceptance of health care

## Abstract

**Aims:**

Lithuania is one of the countries where public and private primary health care (PHC) providers compete for patients. Patients continuously shift from public to PHC providers, but an analysis of the main reasons was never performed. This study aimed to analyze the reasons why patients shift from public to private PHC providers and identify the associations between the reasons and demographic characteristics of the patients.

**Methods:**

A cross-sectional study based on a phone questionnaire was conducted among patients who shifted from public to private primary health care (PHC) providers. A total of 810 phone calls were made, and 572 telephone surveys were completed. The response rate was 70.49%. The difference between the proportions was assessed using the Z-test. The association between categorical variables was assessed using the chi-square test.

**Results:**

The study identified the following main reasons: long queues to obtain family physician appointments (23.6%), inconvenient location of public’s institution department (20.1%), patients relocating (19.2%), enrolment at a former family physician who transitioned from a public to private PHC institution (10.5%), and long waiting time at the family physician’s office for the appointment (9.4%). Some statistically significant correlations were found between the specific reasons for shifting from public to private PHC organizations and patients' demographic characteristics.

**Conclusions:**

Personal reasons are the most common reasons for shifting from public to private PHC providers (43.36% of the respondents), following the reasons related exclusively to the family physician (25.17%) and related PHC institutions only (24.9%).

**What do we already know about this topic?**
There are revealed the main reasons why the patients change their family physicians: a distance to primary health care organization, long waits, problems with the family physician, weak relationship with the family physician, and family physician’s disorganization.
**How does the research contribute to the field?**
There are not so many studies on patients who changed family physician in primary health care, since the patients show high level of satisfaction regarding their family physicians. Most of these studies have focused on the patients shift from public to public or from private-to-private primary health care providers. We conducted a research on patients, who shifted from public to private primary health care provider. To our knowledge this is the first study designed to analyze the reasons of the patients’ shift from public to private primary health care provider.
**What are the research implications toward theory, practice, or policy?**
Our study found that both the primary health care organization and family physician are in control of the majority of the main reasons for patients' shift from public to private primary health care providers. Public primary health care providers should pay more attention to the management of wait times, retain the family physicians at the public PHC organization, and improve the doctor-patient relationships.

## Background

Primary health care (PHC) addresses the majority of a person’s health needs throughout their lifetime.^1^ High-quality PHC systems consistently deliver services that are trusted and valued by the people they serve and improve health outcomes. Within the primary health care performance initiative framework,^2^ five core functions underpin high-quality care delivery in PHC systems: first contact accessibility, coordination, continuity, comprehensiveness, and person-centeredness.^3,4^ There is no doubt that these functions are important. Nevertheless, continuity is a critical but often neglected function of high-quality primary care.^5^ Continuity refers to a long-term healing relationship between a person and his or her primary care provider or care team over time.^4^ There are certain obvious advantages of continuity of care, such as the doctor knowing the patient records (information continuity) and familiar surroundings (organizational continuity),^6^ associated with improved patient, physician, and staff satisfaction, increased adherence to medications and appointments, increased patient disclosure of emotional problems, greater patient trust in physicians, decreased use of laboratory tests, better recognition of medical problems, increased receipt of preventive services and comprehensiveness of care, better coordination of care, improved chronic illness care, decreased preventable adverse medical events, decreased use of emergency rooms, fewer and shorter hospitalizations, and decreased cost of care.^7^

There is a potential trade-off between patient choice and continuity. Continuity could be lost when patients change their family physicians, while other high-quality care core functions remain at the same level. Patients are free to register with any primary care center and family physician in their locality in all European countries, except Finland, Greece, and Sweden, where patients are assigned to a primary care center, and Slovenia, where patients are assigned to a family physician.^1^ Two predominant modes of primary care provision exist across European countries: solo practice and group practice staffed by physicians and other health professionals. Group practices are public primary care clinics and private groups that are staffed by at least one physician and other health professionals (e.g., nurses). Solo practices are private practices in which only one physician works with no other health professionals. In 13 European Union (EU) countries, a solo practice is reported as the predominant form of primary care provision, while in the other 15 EU countries, including Lithuania, a group practice is the predominant model for primary care provision. The patient’s right to choose a family physician is closely associated with changing the family physician according to the patient’s willingness. The majority of EU countries use capitation or fee-for-service payments for primary care^8^; thus, each lost patient causes lost income for PHC organizations, family physicians, or both. In countries where there is competition among PHC providers in a market, patients have free choice of provider, providers compete to attract patients and payment to providers based on patients (capitation),^9^ and the ability to retain the patients is essential.

Some studies discover the reasons for changing family physicians: distance to PHC organization, patient’s problems with doctors, and difficulties with the practice.^7,10,11^ Others emphasize the importance of the doctor-patient relationship in a general practice (GP)^6,12^ and the satisfaction of the patient with their family physician.^13,14^ Nevertheless, these studies focused on the shift from public-to-public^11^ or private-to-private PHC providers.^7,10^

In the Lithuanian PHC sector, both public and private healthcare providers compete for patients. Since 1998, policy proposals have focused on the establishment of private GP practices, which involved public financing of PHC with private family physicians through the National Health Insurance Fund. The development of private GP was supported by certain political decisions (e.g., the application of the same payment rules for private and public providers for value-added tax) and investments.^15^ Each patient in Lithuania is free to choose their PHC provider (health care organization and physician)^16^ and will incur any current or future expenses if the patient has chosen a private PHC provider. Payment for a PHC provider is organized through territorial National Health Insurance Fund branches and based on capitation. Capitation is the predominant form of payment, accounting for 80.7% of the total revenues of PHC organizations in 2018. During the last decade, the proportion of patients enrolled with private PHC providers has increased from 25.46% in 2008 to 31.21% in 2018.^17^ In 2018, current Lithuanian health expenditures accounted for 6.57% of gross domestic product (GDP), the fifth lowest in the EU, and substantially lower than the EU average (9.87%). In 2018, Lithuania spent 1714 euros per person (adjusted for differences in purchasing power), slightly more than half of the EU average (3078 euros per person). Furthermore, only about two-thirds (68.4%) of health expenditures are publicly funded, a significantly lower share than the EU average (84.5%). The number of general practitioners in 2018 was 91.38 per 100 000 of the population, the fourth highest in the EU. A total of 2.2% of the Lithuanian population reported barriers to access to care in 2018 due to waiting time, costs, or distance to travel, while the difference across income groups was relatively small.^18^

The situation in Lithuania, where public and private PHC providers compete for patients, specifically underlines the need to survey the reasons why patients shift from public to private PHC providers.

## Aims

This study aimed to analyze the reasons why patients shift from public to private PHC providers and identify the associations between the reasons and demographic characteristics of the patients.

## Methods

### Settings

This cross-sectional study was conducted at the Kaunas Polyclinic, the largest public PHC provider in Kansas City. In 2018, the Kaunas Polyclinic provided PHC services for 176 961 patients (93.1% of the total number of patients enrolled at the public PHC organizations in Kaunas City) in 5 branches at different geographical locations in the city. The Kaunas Polyclinic also provide secondary specialized outpatient health services.

### Study Design

The survey was carried out among patients who refused PHC services at the Kaunas Polyclinic between January 1, 2018 and December 31, 2018. The survey was conducted by phone. Patients over 18 years old, who refused PHC services at the Kaunas Polyclinic and were enrolled with a private PHC service provider, were included in the study. Patients younger than 18 years old, who refused PHC services at one branch of the Kaunas Polyclinic and enrolled at another branch of the Kaunas Polyclinic, and who were enrolled at another public PHC organization were excluded from the study. Data on adult patients who stopped their PHC services at the Kaunas Polyclinic and enrolled at private PHC providers were assembled from the Polyclinic Information System. The following data were collected: name, gender, year of birth, and contact phone. Patients were selected for telephone calls using a real random number generator (www.random.org). If the patient did not answer the phone call, the call was not repeated. Oral informed consent was obtained during the phone conversation. Between the information-giving and consent stages, the participant had a reasonable amount of time to consider whether to consent. After obtaining oral consent, a written consent form on the participant’s behalf was completed. An oral process was only appropriate due to limited time for consent because the chance for interaction between the researcher and participant depends on conversation time. During the phone questionnaire survey, only one main question was asked to identify the reasons why patients shifted from public to private PHC providers.

**Figure 1.**
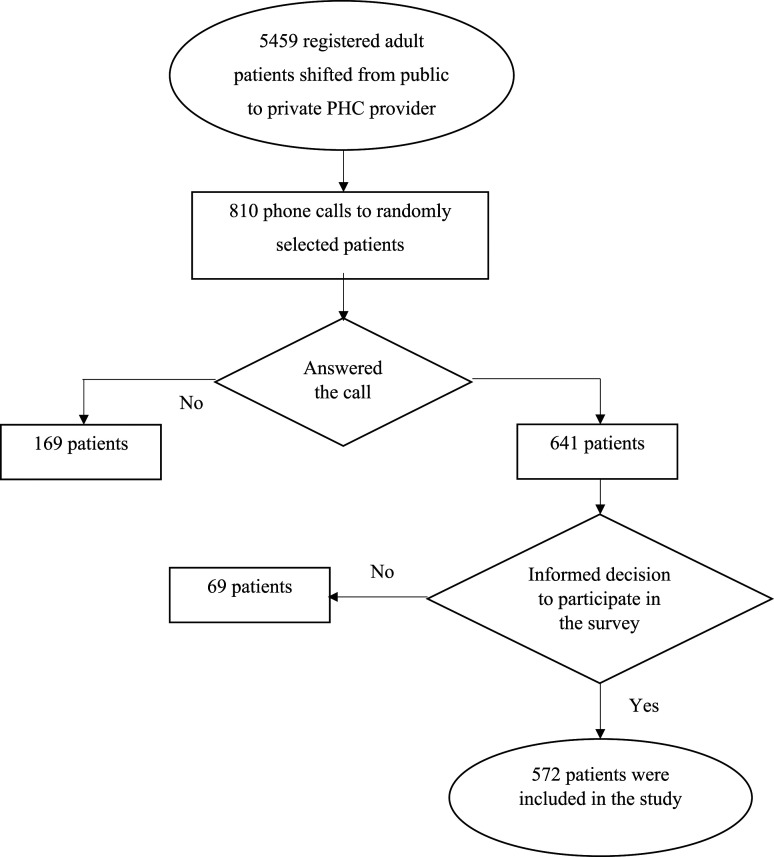
Participants in the study.

A total of 5495 adult patients decided to refuse PHC services at the Kaunas Polyclinic and were enrolled at private PHC providers in 2018. A total of 810 phone calls were made between December 2018 and February 2019. Of the 810 patients, 572 consented to participate in the telephone questionnaire, and 238 did not consent to participate or did not answer the phone call. The response rate was 70.49% (Figure 1).

This study was approved by the Bioethics Committee of Lithuanian University of Health Sciences on 01-03-2018, Protocol No. BEC-VSV(M)-87.

### Statistical Analysis

Statistical analyses were conducted using the SPSS statistical software, version 20.0. The difference between the proportions was assessed using the Z-test. The association between categorical variables was assessed using the chi-square test. The level of statistical significance was set at *P* < .05.

## Results

A total of 568 respondents identified 35 different reasons why they shifted their PHC provider from public to private, which were classified into 4 groups. Four respondents did not specify any reason and were assigned to the first group. Table 1 summarizes the reasons why patients shifted from public to private PHC providers. The first group included only the personal reasons mentioned (248 patients, or 43.36% of the respondents). The second group specified reasons related exclusively to the family physician (144 patients, 25.17%). The third group mentioned reasons relating to PHC institutions only (131 patient or 24,9%). The fourth group mentioned 2 or more reasons related to personal reasons and/or the family physician and/or PHC institution (49 patients or 8.57%).

**Table 1.** Distribution of the Main Reasons to Shift from Public to Private PHC Provider.

Why did you Decide to Shift from Public to Private PHC Provider	No of Patients (n = 572)	%
Group 1. Only for personal reasons	248	43.36
Group 2. Only for reasons relating to family physician	144	25.17
Group 3. Only for reasons relating to public PHC institution	131	22.90
Group 4. Two or more reasons relating to personal reasons, family physician, and public PHC institution	49	8.57

Table 2 lists the detailed reasons of patients why they shifted from public to private PHC providers.

**Table 2.** Frequency of Detailed Reasons to Shift from Public to Private PHC Provider.

Reasons of Giving up the Primary Health Services at the Public Institution	No of Patients	%
Personal reasons		
Inconvenient location of public’s institution department	115	20.1
Moved to live elsewhere	110	19.2
Enrolled at the same private PHC provider as their family members	36	6.3
Turned 18 years old and could no longer use paediatric services	10	1.7
Personal decision without the specific reason	4	0.7
Conflicts with other patients	4	0.7
High risk of getting infected from other sick patients	2	0.3
Enrolled to the family physician at the working place	1	0.2
Accepted discount for some specialized services at the private PHC provider	1	0.2
Reasons relating to family physician		
Enrolled at a former family physician who shifted working place from public to private PHC organization	60	10.5
Treatment failure	32	5.6
Family physician’s insensibility to the patient’s problems	29	5.1
Denial of requested services	24	4.2
Disrespectful behavior of family physician	19	3.3
Distrust with the family physician	12	2.1
Doubts about the competence of family physician	10	1.7
Administrative errors	7	1.2
Provided recommendation to change family physician by others	7	1.2
The age of family physician	5	0.9
Family physician went on maternity leave	5	0.9
Family physician does not spend enough time for me	2	0.3
Family physician was asking for a bribe	2	0.3
Reasons relating to public PHC institution		
Too long queues to get family doctor’s appointment	135	23.6
Long waiting time at the family doctor’s office on the appointment	54	9.4
Better quality of the private PHC provider services	15	2.6
The public organization (department) does not provide required specialized service(s)	10	1.7
Disrespectful behavior of other public organization’s staff	9	1.5
Too long queues to get specialized physician’s appointment	5	0.9
Car parking problems near public’s organization department	5	0.9
Inconvenient registration system for the patient’s appointment	3	0.5
Disrespectful behavior of specialized physician	3	0.5
Specialized physician’s insensibility to the patient’s problems	3	0.5
Not cozy environment of public’s organization department	3	0.5
Too much bureaucracy at the public’s organization department	3	0.5
Lack of confidentiality at the public’s organization department	2	0.3
Inconvenient opening hours of laboratory in the public organization department	2	0.3

There were no statistically significant associations between the respondents’ gender and age and their motives for shifting from public to private PHC providers, except in the case of the reasons related exclusively to family physicians (Group 2) when analyzing the association between respondents’ demographic characteristics and grouped reasons.

Statistically significant differences were found when comparing proportions of the respondents according to gender and specific reasons for transitioning from a public PHC provider to a private one. A higher proportion of females selected the reasons “Inconvenient location of public’s institution department,” “Disrespectful behavior of family doctor,” and “The public organization (department) doesn’t provide required specialized service(s).” compared to males. A statistically significant higher proportion of males specified the reasons “Too long queues to get family doctor’s appointment” and “Long waiting time at the family doctor’s office on the appointment” compared to females. Also, statistically significant differences were found when the proportions of respondents according to age and specific reason for transitioning from a public PHC provider to a private one were compared. A statistically significant higher proportion of patients 66 years old and older named the reasons “Inconvenient location of public’s institution department” and “Family physician’s insensibility to the patient’s problems” compared to younger age groups. A statistically significant higher proportion of respondents 18–34 years old specified the reasons “Moved to live elsewhere” and “Turned 18 years old and could no longer use paediatric services” compared to older age groups; while the higher proportion of 50–65 year old patients enrolled at a former family doctor who transitioned from s public to private PHC organization compared to other age groups.

Statistically significant correlations were found between the specific reasons for shifting from public to private PHC organizations and patients' demographic characteristics. Males were statistically significantly correlated with the following reasons: queues that were too long to get family physicians’ appointments (*P =* .011) and long waiting times next to the family physician’s office (*P =* .016). Females were significantly correlated with the following reasons: the inconvenient location of public organizations (department) (*P =* .049), disrespectful behavior of family physician (*P =* .027), and public organization department does not require specialized physicians (*P =* .009). The younger age of patients was significantly correlated with the following reasons: moved to live elsewhere (*P* < .001), doubts about the competence of a family physician (*P =* .013), public organization department does not have required specialized physicians (*P =* .041), and turned 18 years old and could no longer use pediatric services (*P =* .001). The older age of patients was significantly correlated with the inconvenient location of the public’s organization (department) (*P =* .003), enrolled at a former family physician (*P =* .047), and family physician’s insensibility (*P =* .017).

## Discussion

The main aim of the study was to analyze the reasons why patients shifted from public to private PHC providers. We analyzed the opinion of the patients on why they decided to cease their relations with the public PHC provider and thus interrupt continuity of care at the PHC level. Continuity of care is associated with patient satisfaction,^19^ while the cause of dissatisfaction may be attributed to frequent physician changes.^20^

Patient satisfaction is defined as the degree to which the individual regards the health care service or product or the manner in which it is delivered by the provider as useful, effective, or beneficial.^21^ It is a multidimensional concept that includes patient characteristics (age, gender, socioeconomic status, and patient’s health status), health service provider characteristics (physician and other medical staff, emphasis on staff behavior, attitude to the patient, and communication), and the organizational aspects of health care organizations where the service is provided (emphasis on managing wait times: the period to get an appointment for the service and waiting time at the physician’s office on the appointment).^22-24^ Identifying a sample of general practitioners' patients who are dissatisfied with their care is not easy^10^ because patients indicate high levels of satisfaction with PHC.^25-28^ The method used in this study provides a picture of patients who decided to leave their family physician in a public PHC organization and enrolled in a private PHC organization. The sample identified here might be considered to include the most dissatisfied patients. To avoid a low response rate, we used the phone call method and achieved a 70.49% response rate. The recruited number of participants in the survey represented the population of the Kaunas Polyclinic who transitioned to a private PHC provider.

We found that almost half of the respondents (43.36%) shifted from public to private PHC providers due to personal reasons only, with the highest proportion of patients transitioning due to inconvenient location of public’s organization department (20.1%), relocation (19.2%), and enrolment at the same private PHC provider as their family members (6.3%). This group of reasons cannot be associated with patient dissatisfaction following leaving the family physician at the public PHC organization.

Other respondents have shifted from public to private PHC providers due to reasons related to family physicians (25.17%), reasons related to PHC institutions (22.9%), or 2 or more reasons, including at least one reason related to family physicians or public PHC institutions. The most common reasons related to the family physician were “Enrolment at a former family physician who shifted working place from public to private PHC organization (10.5%),” “Treatment failure (5.6%),” and “Family physician’s insensibility to the patient’s problems (5.1).” The most common reasons related to public PHC institution were as follows: long queues to get an appointment with the family physician (23.6%), long wait time at the family physician’s office on the appointment (9.4%), and “Better quality of the private PHC provider services.” Except for the patients’ enrolment at a former family physician who transitioned from a public to private PHC organization, other reasons could be associated with some kind of dissatisfaction with the service provided at the public PHC institution.

Billinghurst B. and Whitfield M. concluded that patients change their general practitioner due to distance (40.5%), 35% respondents mentioned dissatisfaction with the personal care given by the general practitioner and 36% mentioned dissatisfaction with practice organization. 21.4% respondents lost confidence in doctors, and 13.1% respondents identified long waits.^10^ Buja et al emphasized that the character and personality of the family physician were the main reasons for changing doctors. As for the weakness attributed to their family physician, most people said that the doctor was “too hasty” and the doctor’s scarce accessibility (e.g., “he does not spend enough time and attention on me”). Another reason for changing family physicians was doctors’ disorganization. The patients complained of lengthy waiting times at the doctor’s office, and less frequently, having difficulty contacting the family physician by telephone.^11^ Mold et al studied the reasons older patients change PHC physicians in the USA. They found that when older patients change their PHC physicians, they usually do so involuntarily due to insurance or cost issues, movements to be closer to family, admission to nursing homes, losing the source of transportation or trouble traveling, as well as reasons for physicians themselves (physician left/died/retired).^7^ We noticed that continuity of care is still an important factor, and 10.5% of patients followed their family physicians who transitioned from public to private PHC organizations. These findings support the importance of the doctor-patient relationship in GP.^29,30^

There is a belief among some Lithuanian populations that the private sector is better than the public sector.^31,32^ In our study, 2.6% of respondents identified this particular reason for shifting from public to private PHC institutions. The results of the Albanian study suggest that while the overall quality ratings were similar, private PHC providers were rated better on the quality of basic amenities, confidentiality, and autonomy.^33^ Wang et al^34^ stated that patients in China rate public PHC clinics better in prompt attention, dignity, communication, autonomy, and confidentiality compared to private clinics. Chin et al concluded that the public sector showed better performance in clinical care than the private sector, contrary to common perceptions in Malaysia, despite providing worse consumer quality.^35^ Similar evidence comes from in-patient services as well. Kruse et al^36^ states that most evidence suggests that public hospitals are at least as efficient as or are more efficient than private hospitals in the EU. A recent study in Iran^37^ showed that the patient safety culture score in public hospitals was higher than that in private hospitals, which indicates that health care in public hospitals is better than private hospitals.

A higher proportion of the youngest participant group (18–34 years old) transitioned from public PHC providers due to relocation and losing the ability to use pediatric services after they became adults compared to other age groups. Patients ages 35–49 years old transitioned from public PHC organizations more often than patients in other age groups because they enrolled at their former family physicians who had transitioned from public PHC organizations to private ones. Despite the lack of information of the associations between the reasons of changing family physicians and patients' demographical characteristics in other similar studies, we strongly support the findings in “an opposite” study, which revealed the determinants of patient choice of healthcare providers: there is no such thing as the typical patient - different patients make different choices in different situations.^38^

The main reasons for the shift from public to private PHC providers are more likely to be similar to those discovered in shifting from public-to-public or from private-to-private PHC providers, while the sequence of the reasons is different. The majority of the main reasons for shifting from public to private PHC providers could be managed by the PHC providers, that is, family physicians and PHC organizations. PHC providers should pay more attention to the management of wait times, which include queues to get an appointment with the family physician and wait times at the family physician’s office for a scheduled appointment. Some aspects of working conditions should be taken into account, such as “Treatment failure,” “Administrative errors.” Disrespectful behavior of the family physician, specialist or other staff can also be managed at the institution level. As stated by Ghasemi et al, if conditions favorable to the occurrence of errors exist in the workplace, they will eventually occur.^39^

Another important management aspect is to retain family physicians at the public PHC organization. Patients value a good doctor-patient relationships, and therefore, they will follow the family physician if they change their workplace. Improvements in doctor-patient relationships, including training physicians to develop their communication skills and improve their behavioral manner, balancing of the family physician’s workload, etc. are important aspects as well.

Other reasons for transitioning from public to private PHC providers, such as the location of a public organization, relocation, or enrolment of the family members of the patient to another service provider are not under the control of the PHC provider. PHC organizations can predict the number of such patients but cannot affect it.

This study has several limitations. We only analyzed 2 demographic characteristics (gender and age) and did not analyze socioeconomic characteristics, such as education, occupation, or income of the patient. Any patient in Lithuania, despite gender, age, education, social status, health status, or income is free to change his or her family physician or the PHC institution to another one, either public or private; therefore, we emphasized an exploratory investigation designed to get a better understanding of the patients’ shift from public to private PHC providers. In addition, we did not evaluate the time, how long the patient had a relationship with the family physician, or the health status of the patient. We emphasized an exploratory investigation designed to gain a better understanding of the patient’s opinion on the reasons for leaving a public PHC provider and deciding to enroll at a private one.

## Conclusions

Personal reasons are the most common reasons for transitioning from public to private PHC providers (43.36% of the respondents), following the reasons related exclusively to the family physician (25.17%) and PHC institutions only (24.9%). There were some statistically significant associations between reasons for shifting from public to private PHC providers and patients' gender and age. PHC organizations and family physicians are in control of the majority of the main reasons for patients' shift from public to private PHC providers, except for personal reasons. Public PHC providers should pay more attention to the management of wait times, retain family physicians at the public PHC organization, and improve doctor-patient relationships.
